# Bridging infectious disease vaccines with cancer immunotherapy: a role for targeted RNA based immunotherapeutics

**DOI:** 10.1186/s40425-015-0058-0

**Published:** 2015-04-21

**Authors:** Elias J Sayour, Luis Sanchez-Perez, Catherine Flores, Duane A Mitchell

**Affiliations:** Department of Neurosurgery, UF Brain Tumor Immunotherapy Program, University of Florida, Gainesville, Fl USA; Department of Pathology, Duke University Medical Center, Durham, NC USA; Division of Neurosurgery, Department of Surgery, Duke Brain Tumor Immunotherapy Program, Duke University Medical Center, Durham, NC USA

**Keywords:** RNA, Vaccines, Immunotherapy

## Abstract

Tumor-specific immunotherapy holds the promise of eradicating malignant tumors with exquisite precision without additional toxicity to standard treatments. Cancer immunotherapy has conventionally relied on cell-mediated immunity while successful infectious disease vaccines have been shown to induce humoral immunity. Efficacious cancer immunotherapeutics likely require both cellular and humoral responses, and RNA based cancer vaccines are especially suited to stimulate both arms of the immune system. RNA is inherently immunogenic, inducing innate immune responses to initiate cellular and humoral adaptive immunity, but has limited utility based on its poor *in vivo* stability. Early work utilized ‘naked’ RNA vaccines, whereas more recent efforts have attempted to encapsulate RNA thereby protecting it from degradation. However, feasibility has been limited by a lack of defined and safe targeting mechanisms for the *in vivo* delivery of stabilized RNA. As new cancer antigens come to the forefront with novel RNA encapsulation and targeting techniques, RNA vaccines may prove to be a vital, safe and robust method to initiate patient-specific anti-tumor efficacy.

## Introduction

Tumor-specific immunotherapy is a burgeoning field targeting tumor antigens in the form of peptides, nucleic acids, and cell lysates to induce host-immunity [1]. The exquisite specificity of the immune system and the recent demonstrated efficacy of immune-based treatment of advanced cancers upholds immunotherapy as a promising therapeutic modality; however, the immune correlates necessary to guide successful intervention remain elusive [2-7]. While further testing is necessary, both humoral and cell-mediated immunity are likely paramount for successful cancer immunotherapeutics [4,8-11]. To bypass the complexity of cellular therapeutics, cancer vaccinations have been advanced to recruit adaptive responses, but have suffered from their weak immunogenicity in human clinical trials [11]. This has prompted the development of RNA cancer vaccines designed to mimic infectious challenge, thereby stimulating the innate immune system to augment cellular and humoral immunity [12].

Although some infectious disease vaccines are capable of inducing cell-mediated immunity, humoral immune responses remain the hallmark of effective infectious disease vaccination strategy [13]. These vaccination strategies can be broken down into anti-bacterial immunizations using inactivated prokaryotic proteins, capsular or conjugated polysaccharides, and anti-viral immunizations utilizing inactivated killed virions or live attenuated viral vaccines [14]. A better understanding of infectious vaccine approaches and immunological responses may bolster current RNA cancer immunotherapeutic strategies.

## Review

### Efficacious infectious disease vaccines

Bacterial immunizations for diphtheria, tetanus, pertussis, (collectively known as DTaP- diphtheria-tetanus-acellular pertussis vaccine), all utilize aluminum (alum) containing adjuvants, and demonstrated efficacious and prophylactic immune responses prompting their adoption into the primary vaccination series in children [15]. Inactivated protein antigens from these bacteria are injected with alum salts, initiating inflammation that recruits dendritic cells (DCs) to process bacterial antigens for MHC class II presentation to CD4^+^ T lymphocytes initiating Th2 responses that induce humoral immunity [15-18]. Additionally, polysaccharide antigens have been utilized to induce humoral responses; these include the 23 valent capsular polysaccharide vaccine against pneumococcus (Pneumovax), and the 4 valent polysaccharide vaccine against meningiococcus (MPSV-4) [9]. Since polysaccharide vaccines primarily induce a B-cell-dependent immune response, preventing bacteremia but not fully protecting against pneumococcal or meningococcal infections, conjugation of capsular polysaccharides with a highly immunogenic protein, (i.e. a non-toxic diphtheria toxoid), has been shown to induce B- and T-cell helper responses for more complete protection [19]. This has been employed in the development of conjugated polysaccharide vaccines including Prevnar-13 for pneumococcus, and MCV-4 (Menactra) for meningiococcus [9].

Similar to inactivated bacterial vaccines, viral vaccines induce humoral immunity through a Th2 dependent response; however there is growing evidence that Th1 mediated immunity plays a prominent role in the induction of antigen specific CD8+ T cells after administration of live RNA viral vaccines [19-30]. For example, influenza A is an RNA virus that can be prepared as both a live attenuated, or killed whole-virion vaccine [31,32]. Unlike killed influenza viruses which contain the inactive viral glycoprotein (hemagglutinin) for the induction of humoral immunity, the attenuated influenza virus contains a live single stranded RNA genome [31,32]. This RNA-genome activates plasmacytoid DCs (pDCs) through TLRs inducing type I IFN production, while activating conventional DCs and stromal cells through retinoic acid-inducible gene I (RIG-I) dependent sensors thereby eliciting potent Th1 T cell immunity [31-37]. Additional examples include the yellow fever RNA whole viral vaccine (YF-17D), which activates DCs through concomitant TLR and RIG I activation, inducing prominent type I interferon (IFN) and CD8^+^ T cell responses [22-24]. Similarly, the DNA vaccinia virus for smallpox may stimulate several pathogen recognition receptors (PRRs) eliciting the induction of CD8^+^ T cell expansion, IFN production, and memory formation [24-26,28,30]. Longitudinal analyses of T cell responses responding to the live yellow fever virus and smallpox vaccines demonstrated brisk and specific primary effector CD8+ T cell responses that ultimately differentiated into long-lived highly functional memory cells [38]. Despite these observations, Bacille Calmette–Guérin (BCG), a live attenuated strain closely related to *Mycobacterium tuberculosis,* is one of the only licensed vaccines thought to work primarily through Th1 T-cell responses [29,39]. The immunogenicity of BCG is attributed to TLR recognition of the bacterial cell wall and nucleic acid-sensing PRRs; however, CD8^+^ T-cell responses are highly variable rendering the efficacy of BCG vaccines unreliable in a clinical setting [40-42].

Thus, while infectious vaccine strategies have had tremendous success, notwithstanding BCG, they have historically relied on humoral immune responses. RNA viruses administered as whole viral vaccines, evidenced by attenuated influenza and yellow fever conjugates, can induce combinatorial Th1 and Th2 responses suggesting that a synergistic approach between cellular and humoral immunity may mediate efficacy in malignancies and resistant infectious diseases.

### Cancer immunotherapeutic RNA vaccines

RNA is recognizable by pathogen-associated molecular patterns (PAMPs), which are potent stimulators of PRRs such as TLR receptors 7/8 thereby activating DCs to initiate anti-tumor responses [43-46]. The activation of these antigen presenting cells (APCs) induces Th1 and Th2 type responses through type I IFN signaling via PAMP recognition by PRRs [12].

#### Nucleic acid vaccines

Based on their propensity for inducing both antibody and T cell responses, nucleic acids encoding for cancer antigens are an attractive immunotherapeutic platform for the transfection of APCs [47]. The magnitude of these responses has been lower in DNA vaccines, which are hampered by inadequate delivery mechanisms and mired with constraints of crossing both cell and nuclear membranes [20,47]. The etiology for DNA’s poor immunogenicity remains unclear, but may involve lower expression of DNA-sensing machinery, differing expression patterns of nucleic acid-sensing PRRs or issues related to DNA delivery and processing in different cell types [47-49]. These concerns, along with the potential for oncogenesis through genomic integration, have ignited RNA vaccine research as a promising alternative [47]. RNA vaccines are advantageous since they require only cytoplasm for entry, cannot be integrated into the genome, and are easy to produce and store [50]. RNA can be derived from limited tumor specimens, amplified to generate copious amounts of patient-specific tumor antigens, and be delivered to patients using *ex vivo* priming of autologous DCs or *in vivo* delivery to target APCs [51-53].

#### ‘Naked’ RNA cancer vaccines

The initial experiments with RNA vaccines demonstrated that direct injection of messenger RNA (mRNA) into murine skeletal muscle induced *in vivo* gene expression [54]. These initial observations provided the foundation for later experiments exploring the anti-tumor immunity elicited from mRNA expression of cancer antigens [55]. One of the first mRNA polynucleotide vaccines was constructed using mRNA transcripts encoding for the human carcinoembryonic antigen (CEA), which conferred protective immunity in mice challenged with CEA-expressing tumor cells [55]. RNA transcripts in these experiments were stabilized through 5′ end capping, 3′ end polyadenylation and through incorporation of the human beta-globin 5′ and 3′ untranslated regions (UTRs) [55]. Direct injection of naked mRNA stabilized with beta-globin UTRs has been shown to prime Th2 responses that can be shifted to Th1 responses after administration of GM-CSF in mice [56]. Given the short *in vivo* half-life of RNA from local and systemic administration, other studies have focused on direct injection of RNA into target organs [57-59]. Direct injection of human alpha-1 antitrypsin mRNA into mouse epidermis using gene gun treatment elicited potent antibody responses with increasing titers after subsequent vaccinations [57]. Additionally, gene gun-based immunization using RNA coding for the melanocytic self-antigen TRP2 linked to EGFP demonstrated effective induction of anti-tumor immunity in a murine melanoma model [58]. Other methods of direct injection have included intranodal injection of RNA directly into secondary lymphoid organs inducing anti-tumor immunity in a murine melanoma model [59]. Interestingly, systemic administration of FLT3 ligand prior to intranodal injection of RNA dramatically enhanced priming and expansion of antigen-specific CD8(+) T cells in lymphoid organs, T-cell homing to melanomas, and anti-tumor efficacy [59,60].

Much of this pre-clinical data has led to the translation of naked RNA vaccination into phase I trials for refractory malignancies [61,62]. In a phase I/II trial, total tumor-derived RNA from melanomas, administered intradermally in conjunction with GM-CSF, was safe, but of uncertain efficacy [61]. These patients were vaccinated with autologous amplified tumor mRNA that could be produced in unlimited amounts from a personalized cRNA library representing a tumor specific transcriptome [63]. In a phase I/II trial for patients with renal cell carcinoma (RCC), naked RNA encoding for tumor-associated RCC antigens was co-administered with GM-CSF and was shown to precipitate anti-tumor immunity [62]. These vaccines generated CD8+ and CD4+ immune responses with no severe side effects and induced fifteen stable disease responses out of thirty evaluable patients with RCC [62].

Alternatively, strategies have focused on making ‘naked’ RNA vaccines self-replicating [64-66]. In these strategies, viral structural genes are replaced by mRNAs encoding for cancer antigens, which can be synthesized and replicated by the virus’ non-structural machinery [64,66-69]. Immunizations with a self-replicating RNA immunogen derived from the Semliki forest virus, elicited antigen-specific antibody and CD8+ T-cell responses against model antigens and prolonged the survival of mice with established tumors [64]. In a human phase I trial against colorectal cancer metastases, tumor RNA packaged into alphaviral vectors were capable of efficiently infecting DCs and induced clinically relevant T cell and antibody responses [70]. Despite these attempts, viral engineering of RNA vaccines requires targeted approaches, and contains safety and feasibility issues such as bypassing the induction of vector-specific neutralizing antibodies [70].

Other methods have focused on condensing ‘naked’ RNA to enhance its stability and immunogenicity. RNA can be stabilized through incorporation of polypeptide cations such as protamines which function to condense nucleic acid [46]. These mRNA-protamine complexes have been shown to be a potent immune stimulus activating murine cells through a MyD88, TLR7 dependent pathway and induced anti-tumor immunity in a murine glioma model after direct intratumoral injection [46,71-73]. They have also been shown to provide balanced cell mediated and humoral immunity when co-delivered with naked uncomplexed mRNA [44,74]. In a phase I/II trial for patients with metastatic melanoma, intradermal injection of protamine-stabilized mRNAs coding for melanoma antigens was shown to be safe and feasible with one complete remission [75]. Additionally, the use of protamine condensed RNA has allowed for the development of novel self-adjuvanted vaccines containing nucleotide modifications to mRNA transcripts [44,72]. Whereas protein and peptide-based tumor vaccines require strong adjuvants to induce immunity, non-coding long chain RNA-based adjuvants were shown to induce enhanced immunostimulatory effects over poly(I:C) and may further enhance the immunogenicity of self-adjuvanted mRNA cancer vaccines [76]. Currently, self-adjuvanted mRNA cancer vaccines encoding for commonly expressed tumor associated antigens are in clinical trials for patients with stage IV non-small cell lung cancer (NSCLC) and prostate cancer [77,78].

#### RNA encapsulated vaccines

While the initial attempts using naked RNA have been promising, RNA degradation remains a concern limiting the amount of RNA available for cellular internalization [56]. To protect and deliver RNA to target cells, the delivery mechanism must be safe, feasible, clinically translatable, and amenable to ‘off the shelf’ manufacturing and distribution for the population at large. Based on these criteria, liposomes have been studied as vehicles for RNA vaccines and have been shown to induce antigen-specific cytotoxic T lymphocytes *in vivo* [79]. Since liposomes deliver their contents intracellularly via receptor-mediated endocytosis, the contents of the endosome are frequently shunted into lysosomal degradation pathways prompting the development of pH sensitive liposomes triggering antigen release for MHC processing at low pH levels [80]. These specialized liposomes have been shown to load DCs *in vitro* with mRNA coding for tumor associated antigens, elict cytotoxic T cell responses in murine models, and induce suppression of metastatic spread in a murine melanoma model [81-84]. Liposomes can be combined with polymers to form lipopolyplexes and have been formulated with a polyethylene glycol (PEG)ylated derivative of histidylated polylysine to encapsulate MART1 antigens inducing protective anti-tumor immunity in a murine melanoma model [66,85]. They can also be combined with self-amplifying RNA or protamine condensed RNA [65,86]. Self-amplifying RNA liposomes were shown to elicit antigen-specific interferon-γ-producing CD4+ and CD8+ T-cells in a murine model for respiratory syncytial infection [65]. Similarly, protamine condensed RNA liposomes coding for the model antigen beta-galactosidase induced cytotoxic T cell and antibody mediated immunity in an *in vivo* model [65,86]. In addition to encapsulating novel RNA designs, liposomes can be combined with viral envelopes forming virosomes (liposomes containing viral envelopes) for the encapsulation and delivery of tumor antigens [87]. Fusion-active virosomes have been shown to deliver protein encapsulated ovalbumin (OVA) to DCs for MHC class I presentation at picomolar OVA concentrations [87]. Although liposomes have an attractive safety profile, many of the these approaches remain limited by decreased targeting efficiency, and reticular endothelial elimination thereby mitigating the potency of the immune response [80].

#### Targeting RNA vaccines

Future vaccine strategies will build on the success of previous advances while ameliorating the limitations of the immunogenic but highly degradable naked RNA vaccines, and the limitations of the protected but inefficient delivery of RNA-liposomal vaccines. The safety and stability profile of liposomes is attractive, and the immunogenicity of RNA is appealing for initiating prolific immune responses. Since RNA-liposomes cannot hone to targeted cells, engineering targeting ligands and moieties may be utilized to transfect desired cells. Pre-clinical studies have shown that IgG-coated liposomes are efficiently taken up and presented to T cells by dendritic cells via Ig FcR while mannosylated liposomes enhance uptake and activation of DCs [88,89]. Meanwhile, single chain antibody fragments (scFv) have been developed against DC receptors CD11c or DEC-205 attached to liposome surfaces, demonstrating efficient DC targeting and anti-tumor immunity [90]. Alternatively, DCs can be ex vivo transfected with RNA through liposomal transfection or electroporation before *in vivo* re-introduction [91,92]. These RNA loaded DCs have been shown to stimulate polyclonal T cell responses against antigens expressed in RCC and metastatic colon cancer [53,93,94]. In a phase I trial for metastatic prostate tumors, autologous dendritic cells transfected with prostate-specific antigen RNA stimulated PSA specific cytotoxic T lymphocyte responses [21]. Similarly, in a phase IB study in pretreated advanced melanoma patients, vaccination with monocyte-derived DCs, electroporated with mRNA coding for melanoma-associated antigen combined with immunostimulatory RNAs (isRNAs) (i.e. CD40 ligand, TLR 4 and CD70), induced tumor associated antigen (TAA) CD8+ immunity [95,96]. In these pretreated advanced melanoma patients, two of fifteen patients achieved a complete response and two achieved partial response [95]. While these findings are encouraging, the advancement of *ex vivo* generated cellular vaccines through multi-institutional clinical trials and eventual distribution for widespread clinical utility has proven to be fraught with developmental challenges prompting others to prioritize the advancement of RNA-loaded nanoparticle vaccines as an attractive, “off-the-shelf” targeted therapeutic platform.

#### RNA-loaded nanoparticles

Nanomaterials have been utilized to deliver drugs directly to malignant tissues, bypassing their systemic toxicity [97]. Given the unique physical properties of nanoparticles (NPs) (i.e. size, charge, biocompatibility, solubility), they can be manipulated to increase circulation half-life, accumulation, and drug cargo inside tumors [97]. Nanoscale drug payloads can be associated with targeting ligands and encapsulation techniques to prevent degradation and can be further designed into multi-functional delivery systems with tumor-specific targeting moieties, therapeutic payloads, and diagnostic tools [97-101]. In cancer therapy, NPs have a multitude of various advantages including bypassing multi-drug resistance mechanisms, accessing solid tumors, and engineering the tumor microenvironment [97]. Several pre-clinical studies have investigated the immunogenicity of novel RNA-NP designs. Lipid-like materials, termed “lipidoids,” were shown to deliver immunostimulatory RNA (isRNA) to TLR-expressing cells inducing potent anti-ovalbumin humoral and cell-mediated responses [102]. Similarly, pluronic-stabilized polypropylene sulfide nanoparticles were developed to target antigens in the lung and were shown to deliver their contents to pulmonary DCs inducing potent protective mucosal and systemic CD8(+) T-cell immunity in murine models [103]. Other examples of targeted RNA-nanoparticle immunotherapeutics include mannosylated and histidylated lipopolyplexes loaded with mRNA; these nanoparticles were shown to enhance *in vivo* DC transfection and anti-tumor immunity against a murine melanoma model [104].

Despite this active area of research, the arrival of approved nano-drugs to the market has been slow with only a handful of FDA-approved agents [97,105]. Before nanomaterials can be used in cancer treatments, biodistribution and toxicity must be addressed which depend on the NP size, shape, deformability and surface chemistry (i.e. charge, and pH), all of which are poorly understood in a complex *in vivo* system containing plasma proteins that may alter a nanomaterial’s surface property and affect its biocompatibility [106]. These challenges make each particle, and its toxicity, unique and difficult to investigate as each falls under a diffuse realm of categories including drugs, devices, and biological agents [106-109]. Based on these inherent complications, FDA approval of NPs has stagnated, and most approved agents have only been used in the context of drug delivery where toxicity has traditionally been secondary to the drug as opposed to the biocompatible nanomaterial [109,110].

## Conclusion

Cancer vaccines can meet the challenge of targeted cancer care, but require strong adjuvants to re-direct a ‘tolerant’ immune system. We can look to infectious disease vaccines as a model for successful immunization strategies. While most infectious disease vaccines function through induction of humoral immunity, RNA viral vaccines such as influenza and yellow fever have been shown to generate robust cellular and humoral immunity. To leverage successful cancer immunotherapeutic RNA vaccination strategies, traditional methods in conjunction with novel adaptations are requisite in developing safe and effective formulations that protect RNA from degradation, deliver payload to target, and remain amenable to central manufacturing and distribution. The rapid advancements being made in *in vivo* targeting technologies, particularly in the area of nanomaterials engineering, and our understanding of the requirements for successful induction of potent anti-tumor immunity promise to yield the development of effective and safe RNA vaccines for the treatment of refractory cancers.

## Abbreviations

APC: Antigen presenting cell: APC
Alum: Aluminum
BCG: Bacille Calmette–Guérin
CEA: Carcinoembryonic antigen
DCs: Dendritic cells
HiB: Haemophilus influenzae type b
isRNA: Immunostimulatory RNA
IFN: Interferon
mRNA: Messenger RNA
NSCLC: Non-squamous cell lung carcinoma
OVA: Ovalbumin
PAMP: Pathogen associated molecular pattern
PRRS: Pathogen recognition receptors
pDCs: Plasmacytoid DCs
PEG: Polyethylene glycol
RCC: Renal Cell Carcinoma
RIG-I: Retinoic acid-inducible gene I
TAA: Tumor associated antigen
UTR: Untranslated Regions
